# Increased Serum Soluble Transferrin Receptor Levels Were Associated With High Prevalence of Cardiovascular Diseases: Insights From the National Health and Nutrition Examination Survey 2017–2018

**DOI:** 10.3389/fcell.2022.874846

**Published:** 2022-04-12

**Authors:** Shiyu Zhu, Chang Liu, Chengchen Zhao, Guanzhong Chen, Simin Meng, Ma Hong, Meixiang Xiang, Yao Xie

**Affiliations:** Department of Cardiology, Second Affiliated Hospital, Zhejiang University School of Medicine, Hangzhou, China

**Keywords:** iron deficiency, soluble transferrin receptor, cardiovascular disease, coronary heart disease, heart failure, The National Health and Nutrition Examination Survey

## Abstract

**Background:** Iron deficiency is common in cardiovascular diseases (CVD), e.g., heart failure and coronary heart disease. Soluble transferrin receptor (sTfR) is a promising marker representing unmet cellular iron demands. However, whether higher serum sTfR is associated with increased risk of CVDs needs further investigation.

**Methods:** In the present cross-sectional study, we analyzed data of 4,867 adult participants of the National Health and Nutrition Examination Survey (NHANES) 2017–2018. Linear regression models were employed to identify possible correlations between sTfR and other characteristics. The association between sTfR and CVDs was assessed with univariable and multivariable logistics regression models.

**Results:** The prevalence of CVDs was 9.5% among participants, and higher sTfR levels were found in participants with CVDs (*p* < 0.001). Linear regression models revealed positive associations between sTfR and age, body mass index, systolic blood pressure, glycated hemoglobulin A1c, and insulin resistance (all *p* < 0.001). In the multivariable logistics regression model, the adjusted odds ratio of sTfR for CVDs was 2.05 (per 1 log_2_ mg/L, 95% confidence interval: 1.03∼4.05, *p* = 0.046). Further subgroup analysis identified the associations of sTfR and CVDs were only significant in participants ≥60 years old, or with hypertension (all *p* < 0.05).

**Conclusion:** Our study demonstrated that increased serum sTfR levels were associated with a high prevalence of cardiovascular diseases.

## 1 Introduction

Iron, an essential trace element, involves in multiple cellular biological processes including DNA synthesis, enzymatic activity, and mitochondrial function (Pasricha et al., 2021). Iron metabolism strongly correlates with cardiovascular diseases (CVD). In 1981, J. Sullivan firstly hypothesized that iron overload might contribute to the development of CVD (Sullivan, 1981). However, this hypothesis has not been verified in later clinical studies (Lapice et al., 2013; von Haehling et al., 2015). Iron deficiency (ID), on the contrary, has detrimental effects on cardiovascular diseases including coronary heart disease (CHD) and heart failure (HF). In the Ludwigshafen Risk and Cardiovascular Health Study, ID was found to be associated with CHD, independent of concomitant anemia (Grammer et al., 2014). In patients with HF, ID remained a common complication affecting up to 55% of chronic HF patients and 80% of acute HF patients (McDonagh et al., 2021), along with deteriorated functional status and prognosis (Rocha et al., 2018; von Haehling et al., 2019). Subsequent intravenous iron repletion improved the prognosis of HF patients with ID (Anker et al., 2009; Ponikowski et al., 2015; van Veldhuisen et al., 2017).

Serum ferritin and transferrin saturation (TAST) is currently the mainstay for ID diagnosis (Lopez et al., 2016; Pasricha et al., 2021). However, the accuracy of these iron markers is being questioned under certain medical conditions. Inflammation, liver diseases, and malignancy may increase ferritin concentrations (Thomas et al., 2013; Lopez et al., 2016; Restrepo-Gallego et al., 2020), resulting in a diagnostic sophistication. Transferrin levels may decrease disproportionately to serum iron in case of malnutrition, leading to an elevated TSAT that confuses the diagnosis of ID (Wish, 2006).

Soluble transferrin receptor (sTfR), a truncated monomer of tissue transferrin receptor 1 (TfR1) expressed by almost all proliferating cells, is a promising marker representing unmet cellular iron demands (Beguin, 2003; Restrepo-Gallego et al., 2020). During ID, TfR1 is overexpressed especially in erythroid precursors, which is proportional to the increase in serum sTfR levels (Beguin, 2003). Serum sTfR was proved an indicator of early ID in general population (Baynes, 1996). Most importantly, serum sTfR are not affected by inflammation (Beguin, 2003).

Previous studies have demonstrated the predictive value of sTfR in the prognosis of CHD (Ponikowska et al., 2013; Weidmann et al., 2020) and HF (Jankowska et al., 2014; Biegus et al., 2019; Sierpinski et al., 2021). Conversely, few studies illustrated the association between sTfR and CHD (Braun et al., 2004; Sun et al., 2008), where the relatively small sample size or restricted inclusion criteria affected the validity of these findings. Therefore, in this large-sample cross-sectional study from the National Health and Nutrition Examination Survey (NHANES), we aim to determine whether higher sTfR levels are associated with increased risk of CVDs.

## 2 Methods

### 2.1 Study Population

NHANES is a serial nationally representative cross-sectional survey of the non-military non-institutionalized US population, conducted by the National Center for Health Statistics (NCHS) using a multistage and stratified sampling design. The NHANES collects data from participants through home interviews, as well as physical examinations and laboratory results at the mobile examination center (MEC). Detailed descriptions of NHANES methods are available at the NHANES website (https://www.cdc.gov/nchs/nhanes/index.htm). The NHANES protocols were approved by the NCHS Research Ethics Review Board, and informed consent was obtained from all participants.

**FIGURE 1:**
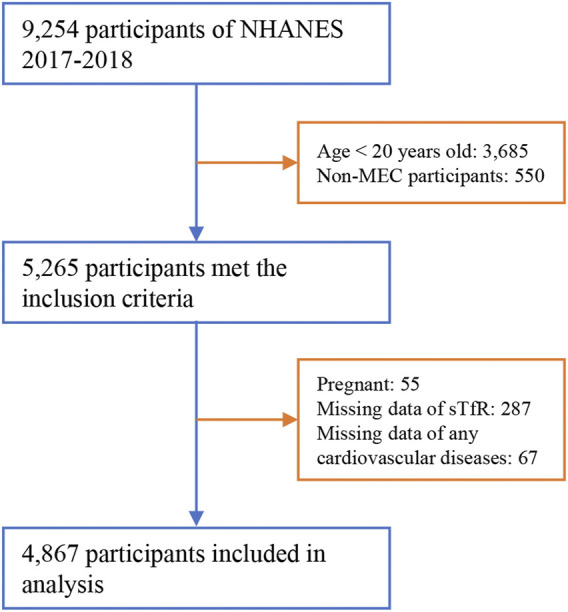
Flow chart of the study.

In this cross-sectional study, we included all adult participants (aged above 20 years old) examined in the MEC from NHANES 2017–2018 (*n* = 5,265). Participants who were pregnant during the survey (*n* = 55), or with missing data of sTfR levels (*n* = 287) and/or any cardiovascular diseases (*n* = 67) were excluded. The entire process of participant inclusion is shown in Figure 1.

### 2.2 Measurement of Serum Soluble Transferrin Receptor Levels

During the MEC examination, blood samples were collected, centrifuged, and stored at <−20°C until analyzed. Serum sTfR levels were measured at the National Center of Environmental Health (NCEH) with the Tina-quant® assay, a particle enhanced immunoturbidimetric assay using Roche kits on the Roche Cobas® c501 analyzer. A detailed description of sTfR measurement is available at https://wwwn.cdc.gov/nchs/data/nhanes/2017-2018/labmethods/TFR-J-MET-508.pdf.

### 2.3 Assessment of Cardiovascular Diseases

Cardiovascular diseases were defined as a composite of self-reported physician diagnoses including heart failure (HF), coronary heart disease (CHD), angina pectoris, myocardial infarction (MI), and stroke, according to previous studies (Abdalla et al., 2020; Cai et al., 2020). Participants would be considered to have CVDs if they responded “yes” to any of the following questions: “Has a doctor or other health professional ever told you that you had congestive heart failure/coronary heart disease/angina (also called angina pectoris)/a heart attack (also called myocardial infarction)/a stroke?” (5 separate questions with the same pattern).

### 2.4 Covariates

Demographic variables (age, gender, and ethnicity) were collected during home interviews. Medical conditions including diabetes, hypertension, hyperlipidemia, drinking (at least 1 alcoholic drink per month or not), and smoking (smoked at least 100 cigarettes during lifetime or not) were obtained by self-reporting. During the MEC examination, height, weight, waist circumference, and blood pressure were measured using standard protocols. Dietary nutrition intake was assessed with the first 24-h dietary recalls, and nutrient values were assigned to foods by using the USDA Food and Nutrient Database for Diet Studies. Laboratory results including blood routine, basic biochemical status, lipid profiles, fasting glucose and insulin (only available in participants examined in the morning), glycated hemoglobin A1c (HbA1c), hypersensitive C-reaction protein (hs-CRP), and other serum iron markers (serum iron, ferritin, and TSAT) were measured with the blood samples obtained during the MEC examination using standard protocols. Low-density lipoprotein-cholesterol (LDL-C) were calculated using the Friedewald equation [LDL-C (mg/dl) = total cholesterol (mg/dl) - high-density lipoprotein-cholesterol (mg/dl)—triglycerides (mg/dl)/5]. Insulin resistance was assessed using the homeostasis model analysis (HOMA), where insulin resistance = glucose levels (mmol/L) × insulin (mU/L)/22.5 (Matthews et al., 1985). Anemia was defined as hemoglobulin <12 g/dl in females or <13 g/dl in males.

### 2.5 Statistical Analysis

Continuous variables were presented as mean ± standard deviation (SD) or median [interquartile range (IQR)] according to the normality of their distribution. Categorical variables were presented as numbers (weighted percentage). Population characteristics were compared among participants with/without CVDs using Student’s t-test or Mann-Whitney *U* test (continuous variables), or χ^2^ test (categorical variables). For further analyses, variables with right-skewed distributions were log_2_-transformed to normalize their distributions. Univariable and multivariable linear regression models were employed to identify possible relationships between sTfR and covariates. The multivariable model was established by forward stepwise selection based on the multivariate *p*-values of each variable. The associations between sTfR levels and the prevalence of total CVDs and individual types of CVDs (HF, CHD, angina pectoris, MI, and stroke) were analyzed using univariable and multivariable logistics regression models. Model 1 was adjusted for demographic variables including age, gender, and ethnicity. In model 2, additional adjustment for traditional cardiovascular risk factors (smoking, BMI, SBP, LDL-C, and HbA1c) was made. Model 3 was adjusted for all model 2 variables plus hemoglobulin. Subgroup analyses were performed according to age, gender, ethnicity, diabetes, and hypertension; and potential interactions were tested. We further compared the association between cardiovascular disease and other iron markers with the univariate and multivariate logistics regression models.

All statistical analyses were performed with the “survey” package (version 4.1-1) for R statistical software (version 4.1.1, R Foundation for Statistical Computing, Vienna, Austria) to take into account the complex survey design of NHANES. Appropriate sampling weights and the Taylor series linearization method were applied in accordance with NCHS recommendations. All statistical tests were two-tailed, and a *p*-value less than 0.05 was considered statistically significant.

## 3 Results

### 3.1 Population Characteristics

Population characteristics of the included participants are summarized in Table 1. The prevalence of CVDs was 9.5%, whereas heart failure, coronary heart disease, angina, myocardial infarction, and stroke were reported by 2.3%, 4.2%, 2.6%, 3.6%, and 3.2% of participants, respectively. Higher serum sTfR levels were found in participants with CVDs [2.84 (IQR: 2.37∼3.44) mg/L vs. 3.41 (IQR: 2.71∼4.21) mg/L, *p* < 0.001]. Significant differences were also found in age, gender, ethnicity, diabetes, hypertension, hyperlipidemia, smoking, drinking, blood pressure, BMI, waist circumference, serum iron, TSAT, hemoglobulin, lipid profiles, HbA1c, and hs-CRP (all *p* < 0.05).

**TABLE 1:** Population characteristic stratified by cardiovascular diseases.

Characteristic	Overall	Non-CVD	CVD	*p*-value
	*n*= 4,867	*n* = 4,282	*n* = 585	
**Sociodemographic**				
Age, year	49 (33, 62)	46 (33, 60)	67 (58, 76)	**<0.001**
Gender				**0.021**
Male	2,368 (48.7%)	2,027 (47.9%)	341 (56.4%)	
Female	2,499 (51.3%)	2,255 (52.1%)	244 (43.6%)	
Ethnicity				**0.014**
Non-Hispanic White	1,704 (62.9%)	1,409 (62.0%)	295 (72.3%)	
Mexican American	657 (8.9%)	611 (9.4%)	46 (3.8%)	
Hispanic	467 (7.0%)	428 (7.3%)	39 (4.1%)	
Non-Hispanic Black	1,115 (10.9%)	978 (10.9%)	137 (10.9%)	
Others	924 (10.3%)	856 (10.4%)	68 (9.0%)	
**Self-reported medical conditions**				
Diabetes	775 (12.0%)	547 (9.2%)	228 (38.9%)	**<0.001**
Hypertension	1,864 (32.7%)	1,424 (28.6%)	440 (72.2%)	**<0.001**
Hyperlipidemia	1,729 (33.4%)	1,378 (30.2%)	351 (64.1%)	**<0.001**
Smoking	2,052 (42.5%)	1,698 (40.8%)	354 (58.8%)	**<0.001**
Drinking	1,995 (55.5%)	1,812 (57.3%)	183 (38.1%)	**<0.001**
**Physical Examination**				
SBP, mmHg	123.87 ± 17.99	123.01 ± 17.41	132.18 ± 21.14	**<0.001**
DBP, mmHg	72.67 ± 11.95	72.94 ± 11.60	70.10 ± 14.75	**0.009**
BMI, kg/m^2^	29.81 ± 7.22	29.67 ± 7.22	31.15 ± 7.09	**<0.001**
Waist circumference, cm	100.86 ± 17.25	100.24 ± 17.30	107.07 ± 15.36	**<0.001**
**Iron Markers**				
Serum iron, μmol/L	15.76 ± 6.48	15.92 ± 6.54	14.29 ± 5.64	**<0.001**
Ferritin, μg/L	106 (52, 191)	105 (52, 191)	113 (57, 198)	0.196
TSAT, %	27.56 ± 11.28	27.75 ± 11.33	25.78 ± 10.69	**<0.001**
sTfR, mg/L	2.87 (2.40, 3.52)	2.84 (2.37, 3.44)	3.41 (2.71, 4.21)	**<0.001**
**Other Laboratory Test**				
WBC, × 10^9^/L	7.39 ± 3.84	7.33 ± 3.66	7.96 ± 5.22	0.075
Hemoglobulin, g/dL	14.21 ± 1.45	14.24 ± 1.43	13.90 ± 1.66	**0.007**
Anemia	510 (6.6%)	404 (5.8%)	106 (14.8%)	**<0.001**
Platelet, × 10^9^/L	245 ± 63	247 ± 62	223 ± 65	**<0.001**
Total cholesterol, mmol/L	4.89 ± 1.04	4.93 ± 1.02	4.52 ± 1.14	**<0.001**
Triglycerides, mmol/L	1.62 ± 1.24	1.60 ± 1.25	1.88 ± 1.09	**<0.001**
HDL-C, mmol/L	1.38 ± 0.40	1.39 ± 0.40	1.29 ± 0.40	0.004
LDL-C, mmol/L	2.76 ± 0.94	2.80 ± 0.92	2.36 ± 1.02	**<0.001**
HbA1c, %	5.69 ± 0.93	5.64 ± 0.88	6.20 ± 1.18	**<0.001**
Fasting glucose, mmol/L	6.12 ± 1.79	6.02 ± 1.60	7.13 ± 2.91	**<0.001**
Insulin (mU/L)	9.25 (6.04, 15.41)	9.02 (5.95, 15.10)	11.72 (6.97, 20.27)	**0.015**
Insulin resistance	2.42 (1.47, 4.25)	2.36 (1.46, 4.19)	3.16 (1.81, 5.80)	**0.007**
hs-CRP, mg/L	1.84 (0.86, 4.25)	1.76 (0.84, 4.06)	2.75 (1.22, 5.63)	**<0.001**
**Dietary Intake**				
Iron, mg/day	12.58 (8.70, 17.63)	12.60 (8.72, 17.77)	12.48 (8.41, 17.22)	0.509

Continuous variables were presented as mean ± sd, or median (interquartile range), categorical variables were presented as n (weighted percentage).

CVD, cardiovascular diseases; SBP, systolic blood pressure; DBP, diastolic blood pressure; BMI, body mass index; sTfR, soluble transferrin receptor; TSAT, transferrin saturation; WBC, white blood cell; LDL-C, low-density lipoprotein-cholesterol; HDL-C, high-density lipoprotein-cholesterol; HbA1c, glycated hemoglobin A1c; hs-CRP, hypersensitive C-reactive protein.

### 3.2 Correlation of Soluble Transferrin Receptor Levels With Covariates

Linear regression models for sTfR were shown in Supplementary Table S1. In univariate analysis, sTfR was positively associated with age (*p* < 0.001). Compared to males, females had higher sTfR levels (*p* = 0.001), whereas higher sTfR levels were found in the non-Hispanic Black (*p* < 0.001) as well as smokers (*p* = 0.021). What’s more, sTfR levels were positively correlated with systolic blood pressure (SBP), BMI, waist circumstance, triglycerides, HbA1c, insulin resistance, and hs-CRP (all *p* < 0.01), while negative correlations were found between hemoglobulin, eGFR, LDL-C, and sTfR (all *p* < 0.05). Interestingly, there was no significant association between sTfR and dietary iron intake (*p* = 0.132). Multivariate linear regression identified gender, ethnicity, SBP, BMI, hemoglobulin, and triglycerides as independent determinants of serum sTfR levels.

### 3.3 Association of Serum Soluble Transferrin Receptor Levels and Cardiovascular Diseases

Table 2 shows the association between serum sTfR levels and the prevalence of CVDs. In the univariate model, the odds ratio (OR) of sTfR (per 1 log_2_ mg/L increase) for total CVDs was 2.37 [95% confidence interval (CI): 1.86∼3.02, *p* < 0.001]. These associations did not change after adjusting for demographic variables, smoking, BMI, SBP, LDL-C, and HbA1c (OR in Adjusted Model 2: 2.21, 95% CI: 1.42∼3.44, *p* = 0.011). After further adjustment of hemoglobulin, the OR of sTfR for total CVDs was 2.05 (95% CI: 1.03∼4.05, *p* = 0.046). For individual types of CVDs, significant associations were found between sTfR and HF, CHD, angina, MI, and stroke in the univariate model (all *p* < 0.01). However, after adjusting for all Model 3 variables, only angina had a significant association with sTfR levels.

**TABLE 2:** Univariate and multivariate logistics regression model of sTfR for CVDs.

Outcomes	Unadjusted model		Adjusted model 1^a^		Adjusted model 2^b^		Adjusted model 3^c^	
	OR^d^ (95% CI)	*p*-value	OR^d^ (95% CI)	*p*-value	OR^d^ (95% CI)	*p*-value	OR^d^ (95% CI)	*p*-value
Total CVDs	2.37 (1.86, 3.02)	**<0.001**	2.51 (1.83, 3.46)	**<0.001**	2.21 (1.42, 3.44)	**0.011**	2.05 (1.03, 4.05)	**0.046**
Heart Failure	2.19 (1.50, 3.19)	**<0.001**	2.12 (1.24, 3.61)	**0.012**	1.94 (0.84, 4.50)	0.086	1.80 (0.46, 7.12)	0.205
Coronary Heart Disease	2.24 (1.74, 2.88)	**<0.001**	2.61 (1.78, 3.83)	**<0.001**	2.34 (1.24, 4.42)	**0.024**	2.13 (0.80, 5.68)	0.080
Angina Pectoris	2.65 (2.03, 3.45)	**<0.001**	3.20 (2.17, 4.72)	**<0.001**	2.89 (1.50, 5.58)	**0.014**	2.88 (1.01, 8.20)	**0.049**
Myocardial Infarction	1.81 (1.29, 2.53)	**0.002**	1.77 (1.06, 2.98)	**0.034**	1.52 (0.69, 3.37)	0.193	1.46 (0.50, 4.25)	0.271
Stroke	2.11 (1.64, 2.72)	**<0.001**	1.86 (1.32, 2.61)	**0.003**	1.59 (0.96, 2.65)	0.062	1.56 (0.68, 3.58)	0.149

a Adjusted Model 1: adjusted for age, gender, and ethnicity.

b Adjusted Model 2: additionally adjusted for smoking, BMI, SBP, LDL-C, and HbA1c.

c Adjusted Model 3: additionally adjusted for hemoglobulin.

d Odds ratio of sTfR, per 1 log_2_ mg/L increase.

OR, odds ratio; CI, confidence interval; sTfR, soluble transferrin receptor; BMI, body mass index; SBP, systolic blood pressure; LDL-C, low-density lipoprotein-cholesterol; HbA1c, glycated hemoglobulin A1c.

We further assessed the association of other iron markers (serum iron, ferritin, and TSAT) and CVDs (Supplementary Table S2). After adjusting for age, sex, ethnicity, smoking, BMI, SBP, LDL-C, HbA1c, and hemoglobulin, none of serum iron, ferritin, or TSAT showed significant association with total CVDs.

### 3.4 Subgroup Analysis

**FIGURE 2:**
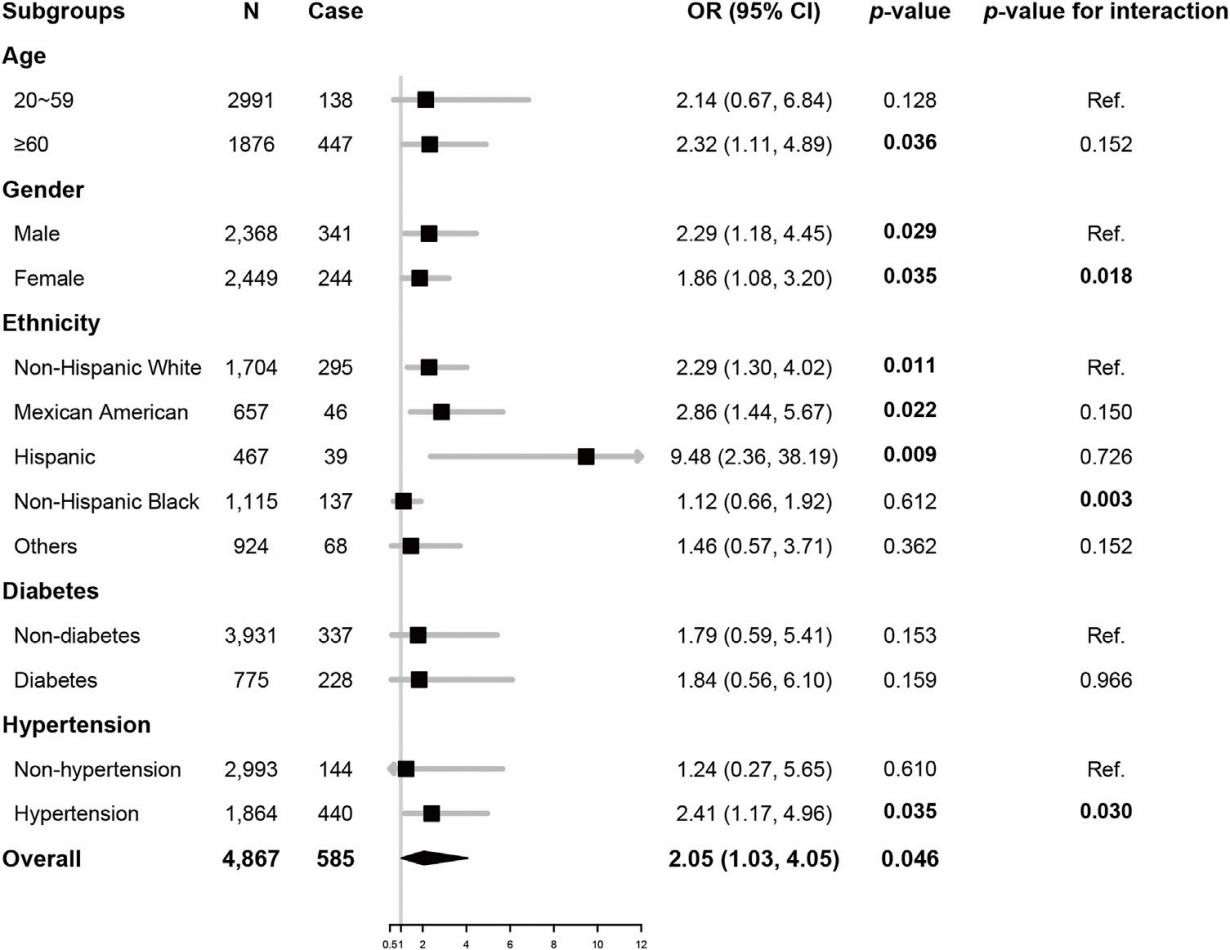
Subgroup analysis Odds ratio of sTfR (per 1 log_2_ mg/L) for total CVDs, adjusted for age, gender, ethnicity, smoking, BMI, SBP, HbA1c, and LDL-C. There were 161 and 10 participants with missing data of diabetes and hypertension, respectively, and data of these participants was excluded from subgroup analysis accordingly. Abbreviations: OR, odds ratio; CI, confidence interval; sTfR, soluble transferrin receptor; BMI, body mass index; SBP, systolic blood pressure; LDL-C, low-density lipoprotein-cholesterol; HbA1c, glycated hemoglobulin A1c.

To further assess the robustness of the association of sTfR and CVDs, subgroup analyses were performed. All analyses were adjusted for age, gender, ethnicity, smoking, BMI, SBP, LDL-C, HbA1c, and hemoglobulin, except for the variable that was stratified. As illustrated in Figure 2, the associations of sTfR and CVDs were only significant in participants ≥60 years old, or the non-Hispanic White, Hispanic, or Mexican American ethnicity, or with hypertension (all *p* < 0.05). Significant associations were also found in both male and female gender (all *p* < 0.05). Potential interactions of sTfR and the stratification factors were also tested, where gender, Hispanic ethnicity, and hypertension had significant interactions with sTfR (all *p* < 0.05).

## 4 Discussion

In this cross-sectional study of a national representing population, we ought to elucidate the association of sTfR and cardiovascular diseases, and our data suggested: 1) higher sTfR levels were associated with higher prevalence for cardiovascular diseases; 2) serum sTfR levels were associated with several cardiovascular risk factors including obesity, hypertension, diabetes, and insulin resistance.

### 4.1 Soluble Transferrin Receptor as an Accurate Marker of Body Iron Status

The gold standard for ID is absent or depicted stainable bone marrow iron (Goddard et al., 2011; Pasricha et al., 2021). Unfortunately, the invasiveness of bone marrow aspiration limits its value in diagnosing iron deficiency. Therefore, ID is diagnosed with serum iron markers such as serum ferritin in most clinical settings. However, in patients with inflammatory status such as atherosclerosis or heart failure (Pearson et al., 2003), the diagnostic value of these markers may be hampered (Restrepo-Gallego et al., 2020).

In normal individuals, cellular iron intake depends on transferrin receptors (TfR) which allows the internalization of the transferrin (Hentze et al., 2004). Serum sTfR, a cleaved monomer of TfR1 identified in 1986, is a relatively novel marker of iron status (Harms and Kaiser, 2015) reflecting the early onset of iron depletion. The most important determinants of sTfR levels are erythropoietic activity and iron status (Beguin, 2003). Serum sTfR levels have been applied in cardiovascular medicine to assist the management of comorbid ID. In patients with CHD or chronic HF, sTfR has been found to have the strongest association with bone marrow ID among serum iron markers (Jankowska et al., 2014; Jankowska et al., 2015; Sierpinski et al., 2021). Most importantly, P. Leszek et al*.* identified sTfR as the only serum marker reflecting myocardial iron load (Leszek et al., 2012).

In contrast to the previous concept that sTfR levels are not affected by inflammation, our study revealed a positive correlation between sTfR and hs-CRP. Similar findings were also shared by several epidemiological studies, where positive correlations were found between sTfR and markers for inflammatory status including hs-CRP (Fernandez-Real et al., 2007; Weidmann et al., 2020) and interleukin (IL)-6 (Sun et al., 2008). Some studies also suggested adjustments should be made for correct interpretations of sTfR in severe inflammation (Thurnham et al., 2015; Rohner et al., 2017). Though sTfR levels may elevate during inflammation, it has been reported that sTfR was less affected by high exposure to inflammation when compared to ferritin (Restrepo-Gallego et al., 2020), which still favored the diagnostic value of sTfR.

### 4.2 Association of Serum Soluble Transferrin Receptor Levels and Cardiovascular Diseases

Our study demonstrated the correlation of sTfR levels and cardiovascular diseases, which was independent of demographic and traditional risk factors. This association was still significant after further adjustment of hemoglobulin, suggesting even early iron depletion might still be associated with cardiovascular diseases regardless of hemopoietic status. Previous research has studied the relationship between sTfR levels and the prevalence of CHD. In a case-control study, higher sTfR levels were found in patients with angiographic CHD, and sTfR levels increased significantly with more coronary arteries affected (Braun et al., 2004). However, in a cohort study of female nurses of the US, there was only a trend toward significance between sTfR and CHD after adjusting for confounding factors (Sun et al., 2008), where the special population might account for this result. What’s more, high sTfR levels independently predict the outcome of patients with CHD, type II diabetes with CHD, or HF (Ponikowska et al., 2013; Biegus et al., 2019; Weidmann et al., 2020; Sierpinski et al., 2021).

### 4.3 Pathophysiological Rationale Linking Soluble transferrin receptor Levels and Cardiovascular Diseases

Our study also revealed the correlation between sTfR and cardiovascular risk factors including obesity as well as central obesity, hypertension, diabetes, and insulin resistance, in line with previous epidemiological studies. Serum sTfR levels were found positively associated with BMI and waist circumference (Freixenet et al., 2009), identical to our findings. In the China Health and Nutrition Survey (CHNS) study, positive associations were found between sTfR levels and both systolic and diastolic pressure at baseline, and high sTfR levels independently predicted the onset of incident hypertension (Zhu et al., 2019). Fernandez-Real JM et al. found that sTfR levels were positively associated with systolic blood pressure, HbA1c, as well as glucose levels during oral glucose tolerance tests (OGTT) in populations with or without diabetes (Fernandez-Real et al., 2007). Furthermore, sTfR was independently and positively associated with insulin resistance measured by homeostasis model analysis in men and postmenopausal women (Suarez-Ortegon et al., 2016). Additionally, Petr Syrovatka et al. found sTfR were positively associated with the sickness of common carotid intima-media in asymptomatic individuals (Syrovatka et al., 2011).

In addition to the associations of sTfR and cardiovascular risk factors, several experimental studies have also revealed potential mechanisms of sTfR or TfR1 involving in CVDs. In atherosclerotic lesions, inflammatory cytokines [e.g., interleukin (IL)-2 and tumor necrosis factor (TNF)] (Tsuji et al., 1991; Seiser et al., 1993), nitric oxide (NO) (Drapier et al., 1993), as well as oxidative stress (Pantopoulos and Hentze, 1995) could upregulate TfR1 expression, which leads to an increased iron intake. Increased iron load in macrophages may contribute to the progression of atherosclerosis by generating reactive oxygen species (ROS) and peroxidation of lipids (Lapice et al., 2013; von Haehling et al., 2015). What’s more, insulin could stimulate cellular iron intake and redistribute TfR1 to cell surface (Davis et al., 1986), which may explain the association between sTfR levels and diabetes as well as insulin resistance.

### 4.4 Study Strength and Limitations

To our knowledge, our study is the first study focusing on the association between serum sTfR levels and cardiovascular diseases with a nationally representing population. Our result strengthened the evidence of the detrimental effects of iron deficiency on cardiovascular diseases. In addition, we examined the relationship between sTfR levels and several cardiovascular risk factors, which were of interest for future studies.

There were several limitations in our study. First, the cross-sectional nature of this study did not allow us to infer a causal association, therefore, further cohort studies or experimental studies are needed to investigate this association. Second, CVDs were ascertained by self-reporting rather than medical records, resulting in recall bias. Third, participants of NHANES were not questioned whether they had hematological disorders (e.g., aplastic anemia or leukemia), therefore we did not exclude participants with hematological disorders. As serum sTfR levels are influenced by erythropoietic activity, hematological disorders might be a potential confounder even if we have adjusted for hemoglobulin in the Adjusted Model 3. Third, we only included data of NHANES 2017–2018 due to data availability, resulting in a relatively low statistical power in subgroup analysis or logistics regression models for individual types of CVDs, raising the concern for false negatives. Furthermore, the relatively high cost and lack of a standard cutoff limits the application of sTfR (von Haehling et al., 2015; Restrepo-Gallego et al., 2020), especially in developing countries.

## 5 Conclusion

In conclusion, our study demonstrated that increased serum sTfR levels were associated with a high prevalence of cardiovascular diseases. Serum sTfR levels, a promising marker measuring unmet cellular iron demand, may have predictive value for risk stratification of CVDs.

## Data Availability

Publicly available datasets were analyzed in this study. This data can be found here: https://wwwn.cdc.gov/nchs/nhanes/continuousnhanes/default.aspx?BeginYear=2017.
