# Supplementary material for: Increased Serum Soluble Transferrin Receptor Levels Were Associated With High Prevalence of Cardiovascular Diseases: Insights From the National Health and Nutrition Examination Survey 2017–2018
Source: Front Cell Dev Biol. 2022 Apr 12;10:874846. doi: 10.3389/fcell.2022.874846 (PMC9039157; doi:10.3389/fcell.2022.874846)
Supplement: Supplementary file 1 [file Table1.DOCX]

Supplementary Material

**Supplementary Table S1** Univariate and Multivariate Linear Regression for sTfR (per 1 log_2_ mg/L)

|  | **Univariate Linear Regression** | | | **Multivariate Linear Regression** | | |
| --- | --- | --- | --- | --- | --- | --- |
| Characteristic | Beta | 95% CI | p-value | Beta | 95% CI | p-value |
| Age, year | 0.002 | 0.001, 0.003 | **<0.001** |  |  |  |
| Gender, female vs male | 0.082 | 0.039, 0.124 | **0.001** | -0.113 | -0.174, -0.051 | **0.005** |
| Ethnicity |  |  |  |  |  |  |
| Non-Hispanic White | Ref. | Ref. |  | Ref. | Ref. |  |
| Mexican American | -0.033 | -0.087, 0.022 | 0.216 | -0.068 | -0.136, 0.001 | 0.051 |
| Hispanic | 0.024 | -0.073, 0.122 | 0.598 | -0.025 | -0.129, 0.079 | 0.566 |
| Non-Hispanic Black | 0.312 | 0.255, 0.370 | **<0.001** | 0.166 | 0.092, 0.240 | **0.002** |
| Others | 0.073 | -0.010, 0.157 | 0.079 | 0.054 | -0.036, 0.143 | 0.184 |
| Smoking, yes vs no | -0.066 | -0.120, -0.011 | **0.021** |  |  |  |
| Drinking, yes vs no | -0.115 | -0.159, -0.072 | **<0.001** |  |  |  |
| SBP, mmHg | 0.003 | 0.002, 0.005 | **<0.001** | 0.002 | 0.001, 0.003 | **0.014** |
| DBP, mmHg | 0.000 | -0.002, 0.002 | 0.992 |  |  |  |
| BMI, mmHg | 0.013 | 0.010, 0.015 | **<0.001** | 0.010 | 0.006, 0.014 | **0.002** |
| Waist circumference, cm | 0.005 | 0.004, 0.006 | **<0.001** |  |  |  |
| WBC, 10^9^/L | 0.004 | -0.001, 0.009 | 0.091 |  |  |  |
| Hemoglobulin, g/dL | -0.103 | -0.123, -0.083 | **<0.001** | -0.111 | -0.140, -0.082 | **<0.001** |
| Platelet, 10^9^/L | 0.000 | 0.000, 0.001 | 0.160 |  |  |  |
| Total cholesterol, mmol/L | -0.015 | -0.032, 0.002 | 0.081 |  |  |  |
| Triglycerides, mmol/L | 0.024 | 0.011, 0.037 | **0.003** | 0.026 | 0.009, 0.042 | **0.010** |
| HDL-C, mmol/L | -0.081 | -0.122, -0.039 | **<0.001** |  |  |  |
| LDL-C, mmol/L | -0.023 | -0.045, 0.000 | **0.047** |  |  |  |
| HbA1c, % | 0.047 | 0.035, 0.059 | **<0.001** |  |  |  |
| Insulin resistance | 0.054 | 0.038, 0.070 | **<0.001** |  |  |  |
| hs-CRP, log_2_ mg/L | 0.049 | 0.034, 0.065 | **<0.001** |  |  |  |
| Dietary iron intake, mg/day | -0.002 | -0.004, 0.001 | 0.132 |  |  |  |

Abbreviations: CI, confidence interval; Ref, reference; SBP, systolic blood pressure; DBP, diastolic blood pressure; BMI, body mass index; WBC, white blood cell; HDL-C, high-density lipoprotein-cholesterol; LDL-C, low-density lipoprotein-cholesterol; HbA1c, glycated hemoglobin A1c; hs-CRP, hypersensitive C-reactive protein.

**Supplementary Table S2** Association of sTfR, Serum Iron, Ferritin, and TSAT with Cardiovascular Diseases

| **Iron Markers** | **Unadjusted Model** | | **Adjusted Model 1^$^** | | **Adjusted Model 2^#^** | | **Adjusted Model 3^&^** | |
| --- | --- | --- | --- | --- | --- | --- | --- | --- |
|  | OR* (95% CI) | *p*-value | OR* (95% CI) | *p*-value | OR* (95% CI) | *p*-value | OR* (95% CI) | *p*-value |
| **sTfR**, per 1 log_2_ mg/L | 2.37 (1.86, 3.02) | **<0.001** | 2.51 (1.83, 3.46) | **<0.001** | 2.21 (1.42, 3.44) | **0.011** | 2.05 (1.03, 4.05) | **0.046** |
| **Ferritin,** per 1 log_2_ μg/L | 1.07  (0.99, 1.16) | 0.103 | 0.91 (0.81, 1.01) | 0.067 | 0.94 (0.77, 1.14) | 0.385 | 0.99 (0.75, 1.30) | 0.851 |
| **TSAT,** per 1% | 0.98 (0.98, 0.99) | **<0.001** | 0.97 (0.96, 0.99) | **0.002** | 0.98 (0.96, 1.00) | 0.081 | 0.99 (0.96, 1.02) | 0.189 |
| **Serum iron,** per 1 μmol/L | 0.96 (0.94, 0.97) | **<0.001** | 0.94 (0.91, 0.97) | **0.003** | 0.95 (0.91, 1.01) | 0.065 | 0.96 (0.90, 1.03) | 0.138 |

^$^Adjusted Model 1: adjusted for age, gender, and ethnicity.

^#^Adjusted Model 2: additionally adjusted for smoking, BMI, SBP, LDL-C, and HbA1c.

^&^Adjusted Model 3: additionally adjusted for hemoglobulin.

Abbreviations: OR, odds ratio; CI, confidence interval; sTfR, soluble transferrin receptor; TSAT, transferrin saturation; BMI, body mass index; SBP, systolic blood pressure; LDL-C, low-density lipoprotein-cholesterol; HbA1c, glycate hemoglobulin A1c.
